# Inter-digital capacitors with aerosol-deposited high-K dielectric layer for highest capacitance value in capacitive super-sensing applications

**DOI:** 10.1038/s41598-018-37416-7

**Published:** 2019-01-24

**Authors:** Eun-Seong Kim, Jun-Ge Liang, Cong Wang, Myung-Yeon Cho, Jong-Min Oh, Nam-Young Kim

**Affiliations:** 1 RFIC Center, Kwangwoon University, Seoul, 139-701 Republic of Korea; 2 School of Electronics and Information Engineering, Harbin Institute of Technology, Harbin, 150001 China; 3 Department of Electronic Materials Engineering, Kwangwoon University, Seoul, 139-701 Republic of Korea

## Abstract

Inter-digital capacitors (IDCs) with aerosol-deposition (AD) high-k dielectric layer were compared via simulation and measurements of bare IDCs and AD IDCs at room temperature and subjected to a post-annealing process for realizing capacitive super-sensing applications. IDCs with thin AD films can provide higher capacitive intensity and improvements for other dielectric performances. Therefore, IDC patterns with AD high-k dielectric layers were fabricated by varying the finger widths and gap. Moreover, we analyzed the layer microstructure design patterns using simulations and experiments with AD BaTiO_3_ as-deposited IDCs and IDCs subjected to annealing at 500 °C. These three different IDCs were measured using an impedance analyzer; furthermore, the AD BaTiO_3_ films were evaluated using X-ray diffraction, atomic force microscopy, and traveling electron microscopy. The results for the IDCs with the AD BaTiO_3_ film show the highest capacitance when compared with other thin layer capacitors, which is expected to be useful in realizing super-sensing applications in the future.

## Introduction

In recent years, the increasing demand for mass production, miniaturization, and high-density integrated capacitors in communication systems has led to the proliferation of thin-film integrated passive devices (IPDs)^1^, high electron mobility transistors (HEMTs)^2^, and digital as well as mixed signal applications^3^. Because passive capacitive components occupy approximately 60% of the area in a typical integrated circuit, it is important to miniaturize these components. Furthermore, to fabricate materials with high relative permittivity, semiconductor technologies, such as sputtering and low-temperature co-firing ceramics (LTCC) technology, can be used^4^; however, both sputtering and LTCC technology require sintering at approximately 850 °C to form the desired crystallite structure during the fabrication process. Considering this, both high temperatures and thermal energy costs related to sintering can be critical limitations for embedding passive devices on integrated circuits. To overcome these problems, recently, the aerosol-deposition (AD) method was developed for the preparation of ceramic films at room temperature^5^. We focus on AD and annealing properties which has a potential for sensing application. In the AD fabrication, we can control the density and that leads to excellent humidity sensor and can be used in other application.

Aside from low-temperature fabrication and reduced energy costs, previous studies have confirmed other advantages of using the AD method, including efficient film growth, controllable thickness from 1.5–3.0 μm for ceramic films, which yields excellent sensitivity and microstructures, and long-shield raw materials, which are useful in industrial fabrication^6^. Based on the results of our previous study related to the effect of annealing on sensitivity, thermal treatment at 100 to 600 °C showed that the best capacitance was achieved at 400–500 °C^7^. Thus, based on these results, it can be deduced that the thickness and annealing of BaTiO_3_ in the AD method can further improve high-k capacitance values.

Inter-digital circuits (IDCs) for humidity sensing are common among conventional capacitive sensors; these sensors are based on the principle of dielectric changes in thin films upon water vapor uptake. Though the properties of these sensors primarily depend on the hygroscopic materials used, electrode geometry plays a key role as well. In a previous research, Korvink considered four different electrode geometries for capacitive humidity sensors^8^ with different electrical field distributions in the dielectric layer. In addition, modeling for multi-layer conductor-facing IDC structures has been studied to predict the capacitive performance during the design step; in particular, the closed form analytical expression derived from the Schwarz-Christoffel conformal mappings were used to determine the capacitance of the sensors^9^. Furthermore, Hong-Ki developed an IDC using the AD method and studied the optimized film thickness through both simulation and fabrication^10^; nevertheless, to the best of our knowledge, efficient design and simulation parameters have not been identified thus far.

In this study, we deposited a BaTiO_3_ film with thickness in the range of 1.5–3.0 μm on the glass substrate of a Pt IDC; in particular, we used BaTiO_3_ powder, which is suitable for the AD method. As previously mentioned, studies have been conducted on thickness control and annealing temperature for such films; however, in the current study, we focused on the effect of different widths and number of electrodes in the AD process and the annealing properties to achieve a higher capacitance with the same thickness. In particular, we measured three different IDC patterns under three different conditions; the first condition was of a bare-chip, while the second and third conditions were the BaTiO_3_ deposition at room temperature and annealing of the BaTiO_3_ film at 500 °C, respectively. Through computer simulation and measurement of the IDC under the three aforementioned conditions, we confirmed that after the BaTiO_3_ AD process annealing 500 °C performs two times higher increasing rate from AD process.

## Methods

**Figure 1:**
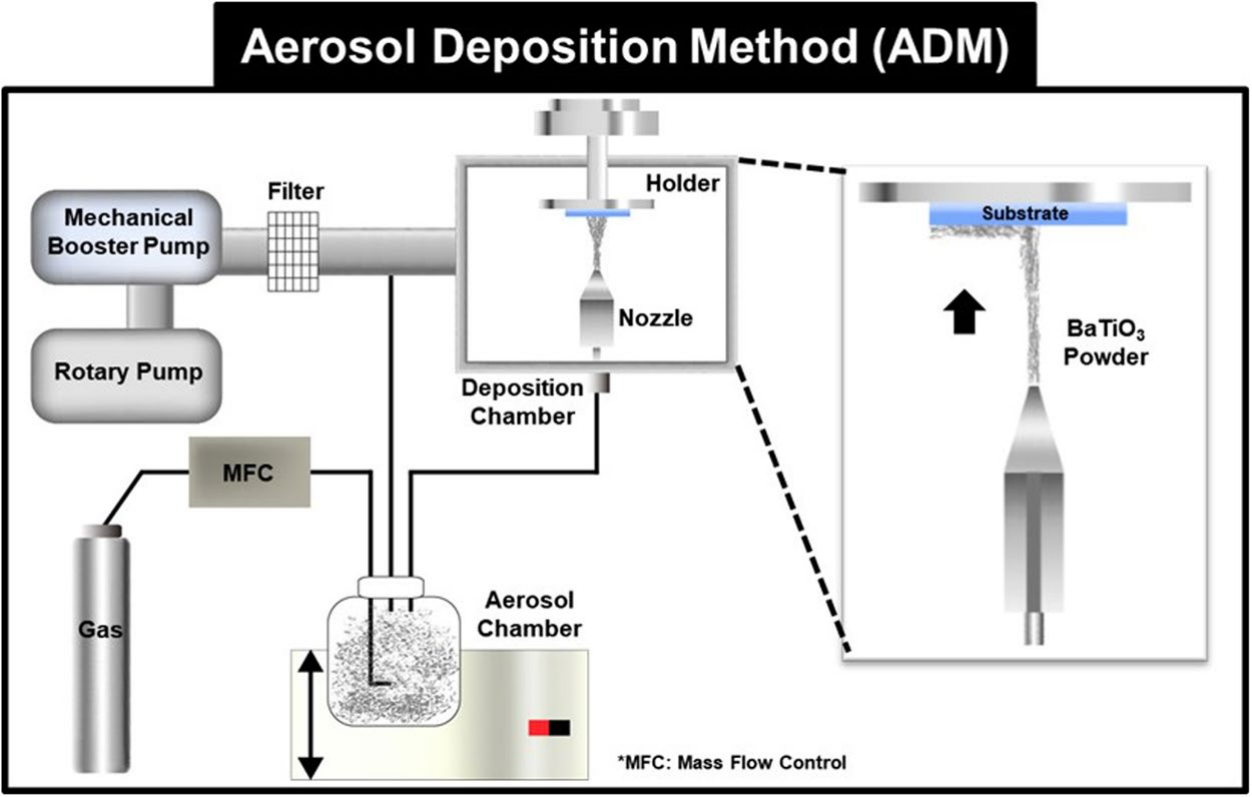
AD high-K dielectric layer preparation method of the AD apparatus and the process of room-temperature growth of the BaTiO3 thin films. For aerosol generation, helium gas was passed through BaTiO3 powder contained in a vibrating chamber, thereby producing a fluidized bed. Driven by a pressure difference, the aerosolized particles are carried from this chamber to the deposition chamber. The BaTiO3 aerosol is accelerated by an aerosol jet at its nozzle orifice.

**Figure 2:**
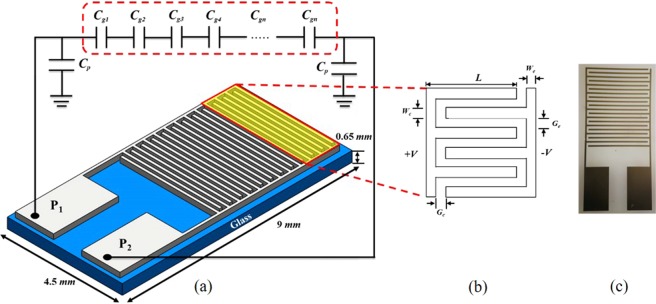
Schematic design of proposed IDC fabricated on glass wafer with the detailed dimensions. (**a**) Type-3 IDC design fabricated by Pt and its equivalent circuit approximating to the cascade of a series of individual capacitors; (**b**) IDC design with width and length dimension markers, and (**c**) IDC graph by microscopic camera.

In our study, three IDCs with different finger widths and equal gaps were designed and simulated using the Advanced Design System (ADS) (2016.01, Keysight©). In particular, the AD method is a room-temperature process that is based on shock-loading solidification of accelerated particles on a substrate, where the particles are transferred by high-speed gas flow. Helium was used to increase the gas flow speed and particle impact velocity of the BaTiO_3_ aerosol, as it is lighter compared with other suitable gases^11^. The chamber gas flow rate was maintained at 4.5 L/min at an interior pressure of 1–7 torr; Gas flow control is important when the powder collides to the substrate strongly in higher flow rate, film will be more denser at the annealing process, but causes deterioration in the inner structure by leading to decreasing capacitance. The BaTiO_3_ particles were deposited on the glass substrate at a scanning speed of 1–2 mm/min. The detailed AD process is shown in Fig. 1 to explain the clear state of the AD method. The other parameters necessary to ensure a final deposited thickness of 1.5 μm, such as the size of the nozzle orifice, distance between the nozzle and substrate, and the operation time, are listed in Table 1. The high-speed impact of BaTiO_3_ particles on the glass substrate causes the particles to partially melt and bond; this causes the crystal lattice to distort and the residual stress significantly affects the dielectric properties of the generated BaTiO_3_ layers. Post thermal treatment has been shown to be effective in overcoming the issues of crystal lattice distortion and residual stress. Thus, in our study, we performed post-annealing on the proposed films at 500 °C. Furthermore, we proposed three IDC designs with different finger widths. The design parameters of our IDCs include the number of electrodes *N*, electrode width *W*_*c*_, electrode length *L*, and gap between the meander line *G* are shown in Table 2, which together determine the capacitance along with the properties of the adopted substrate. The three-dimensional (3D) plot, equivalent circuit, and geometry of our IDC with dimension markers are shown in Fig. 2.

**Table 1:** Typical parameters and conditions for AD experiments in this study.

Parameter/Conditions	Value/Substance
Starting Powder	BaTiO_3_: 450 nm
Substrate	Glass
Size of Nozzle Orifice	10 × 0.4 mm^2^
Scanning Speed	1–2 mm/s
Working Pressure	1–7 Torr
Carrier Gas	He
Carrier Gas Consumption	4.5 L/min
Substrate-to-nozzle Distance	5–10 mm
Deposition Temperature	Room Temperature
Deposition Time	10–30 min
Deposition Area	4.6 × 5.5 mm^2^
Post-annealing Time	2 h
Post-annealing Atmosphere	Ambient
Rate of Temperature Increase and Decrease	5 °C/min
Duration of Temperature Increase and Decrease	1 h 30 min

**Table 2:** Dimensions of the fabricated IDC including the number of electrodes (N), electrode width (*W*_*c*_), side-edge width (*W*_*e*_), distance from electrode to side-edge (*G*_*c*_), distance between adjacent electrodes (*G*_*e*_), and electrode length (L).

C	N	*W*_*c*_ [μm]	*W*_*e*_ [μm]	*G*_*c*_ [μm]	*G*_*e*_ [μm]	*L* [mm]
Type 1	19	150	100	100	100	3.8
Type 2	24	100	100	100	100	3.8
Type 3	32	50	100	100	100	3.8

The annealing setup was set to increase the temperature by 5 °C/min for one and a half hours in air atmosphere. Rapid heating will easily cause serious cracks, which is why we chose to operate this process in a slow manner to reduce the thermal stress generated from the AD process. After heating to the desired temperature, annealing was performed for 2 h, which is the perfect process for a film to be annealed. Then, the temperature was decreased slowly by 5 °C/min for approximately one and a half hours. The AD process works mainly by the hammering effect, which makes the bottom layer dense and top layer highly porous. Meso-cracks will be formed when the annealing temperature is increased suddenly or is increased beyond 500 °C. Our goal was to achieve higher capacitance with the highest quality by annealing at 500 °C, which causes almost no thermal stress or meso-cracks that lower the capacitance. According to AD literatures, carrier gas consumption is one of the important factors for changing the film properties. Actually, prior to fixing the carrier gas consumption of 4.5 L/min in our manuscript, we performed the BaTiO_3_ deposition by using gas consumption of 10 L/min, as shown in the graph below. In this case, although the gas consumption of 10 L/min showed higher capacitance than that of 4.5 L/min, the capacitances were drastically decreased by thermal treatment at 500 °C post annealing. This reason can be contributed to excessively high speed of particles from the nozzle. When the powder collides with the substrate more forcefully, the as-deposited film has a denser structure under room temperature, resulting in high capacitance. However, the residual stress under gas consumption of 10 L/min was very high because of the aforementioned reason, which causes deterioration of the internal structure after annealing at 500 °C, leading to decreased capacitance. On the other hand, under gas consumption of 4.5 L/min, the capacitance was not changed after the AD process as well as after annealing at 500 °C owing to the lower residual stress in the inner structure. Results showed that gas consumption of 4.5 L/min with annealing at 500 °C yielded higher capacitance than the gas consumption of 10 L/min. Therefore, this study concluded that 500 °C is the most ideal annealing temperature for achieving better film quality, which is an important factor for achieving desirable electrical properties.

The impedance analyzer was utilized to modify the capacitance, impedance, dielectric loss in the 1-MHz phase test to explore the mechanism of AD for high-performance capacitor preparation; in addition, the microstructure information of the prepared AD film was studied. An atomic force microscope (AFM) (XE150, PSIA, USA) in the non-contact mode was employed to characterize the surface morphology of the obtained samples. Using the XEI software (Park Systems Corp.), the obtained data were analyzed in terms of the AFM top view, 3D-morphology view, and two-dimensional (2-D) Fourier filter transform (FFT) power spectra. Moreover, a scanning electron microscope (SEM) (S-4800, Hitachi, UK) was employed for observing the cross-section microstructure of the deposited films. Geometric parameters for the IDC were *W*_*e*_ = 100 μm, *W*_*c*_ = 50–150 μm (Type1–3), *G*_*e*_ = 100 μm, *G*_*c*_ = 100 μm, and *L* = 3.8 mm.

## Results and Discussion

### IDC Design Circuit Simulations

**Figure 3:**
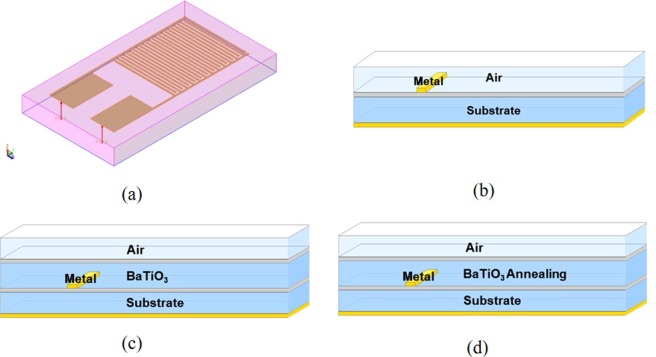
Simulation process of an IDC on a glass substrate in air using ADS (Keysight Technologies). **(a**) 3D view of the designed patterns, (**b**) layer arrangement for the bare chip, (**c**) the design with BaTiO3 films on top of the IDC, (**d**) thermal treatment of the chips.

Before fabrication of the IDCs, we performed simulations using the ADS 2016.01 to predict the capacitance of the prepared IDCs in both bare chips as well as AD films. IDC plays an important role in capacitance and in sensor. We have used IDC design to find ceramic film properties of BaTiO_3_ by the AD process and annealing. Figure 3 shows the cross section to simulate the desired data of the bare chip, room temperature BaTiO_3_, and annealing, using three different dielectric constant and dielectric loss. The simulated frequency was set to 1 MHz. In addition, as previously mentioned, the dummy glass substrates in both the AD and post annealing methods were used to achieve different permittivity and dielectric loss values by simulating three different designs. The relative permittivity $\varepsilon$ of each layer can be estimated using the Hashin-Shtrikman bounds theory; these permittivity values should be different for each layer^12^. The relative permittivity of the layers depends on the grain connectivity ratio (%) and top and bottom limits, which are calculated using Equations (1) and (2) as follows.

1 $${\varepsilon }_{Top}={\varepsilon }_{BTO}{V}_{BTO}+{\varepsilon }_{Air}{V}_{Air}-\frac{{V}_{BTO}{V}_{Air}{({\varepsilon }_{BTO}-{\varepsilon }_{Air})}^{2}}{{\varepsilon }_{BTO}{V}_{Air}+{\varepsilon }_{Air}{V}_{BTO}+(d-1){\varepsilon }_{BTO}}$$

2 $${\varepsilon }_{Bottom}={\varepsilon }_{BTO}{V}_{BTO}+{\varepsilon }_{Air}{V}_{Air}-\frac{{V}_{BTO}{V}_{Air}{({\varepsilon }_{BTO}-{\varepsilon }_{Air})}^{2}}{{\varepsilon }_{BTO}{V}_{Air}+{\varepsilon }_{Air}{V}_{BTO}+(d-1){\varepsilon }_{Air}}$$

The permittivity reduction of one layer to the next is assumed to act as an electric field barrier; in particular, the layers are cascaded in a parallel-type configuration and Neumann boundary conditions are assumed at the layer interfaces. However, in the case wherein the permittivity is increasing from one layer to the next and the electric filed is more strongly guided away from the electrode plane, the layers are assumed to be coupled in series; in this case, Dirichlet boundary conditions are assumed at the layer interfaces^9^. Hence, the capacitances of the bilayer structure film can be calculated for the 20% and 90% relative humidity (RH) conditions using the following equations:

3 $$Parrallel:{C}_{RH}=L[({\varepsilon }_{m1}-{\varepsilon }_{m2}){K}_{film}^{c}(\eta ,{r}_{1})+{\varepsilon }_{m2}{K}_{film}^{c}(\eta ,{r}_{2})].$$

4 $$Series:\frac{1}{{C}_{RH}}=\frac{1}{L}[(\frac{1}{{\varepsilon }_{m1}}-\frac{1}{{\varepsilon }_{m2}})\frac{1}{{K}_{film}^{c}(\eta ,{r}_{1})}+\frac{1}{{\varepsilon }_{m2}}\frac{1}{{K}_{film}^{c}(\eta ,{r}_{2})}].$$

### Crystalline Growth

The first step in the study of BaTiO_3_ film growth involves studying the effect of the shock-loading solidification of BaTiO_3_, whereas the second step involves studying the hammering effect of particle deposition at high speeds; first, these effect causes fragmentation followed by mutual bonding of the BaTiO_3_ particles. In particular, the fragmentation and mutual bonding lead to reduced grain size, induce crystal lattice distortion, and cause residual stress accumulation, which, in turn, affect the crystal structure properties, including the crystallinity and dielectric properties^13,14^.

**Figure 4:**
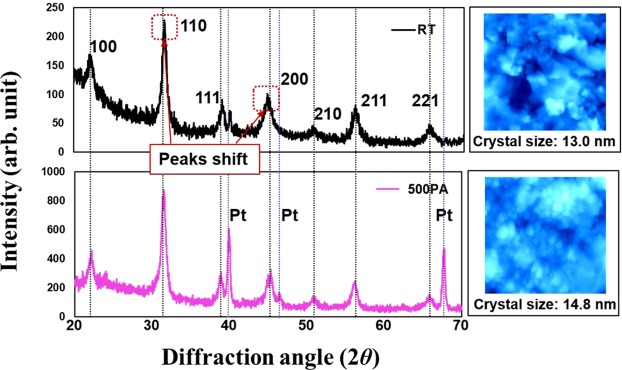
XRD plots accompanied by 2 μm × 2 μm AFM images of AD-prepared BaTiO3 films. (a) The expansion of diffraction peaks at 2θ = 45.37° corresponding to (200) plane revealed a peak shift of 0.69° in the films prepared at room temperature (RT). (b) The heights of the BaTiO3¬ peaks increased along with the eased peak shift and the crystal size increased at high temperature (500 °C) post annealing (PA).

Figure 4 shows the X-ray diffraction (XRD) pattern of the as-deposited BaTiO_3_ film, which reveals its cubic perovskite crystal structure. On post-annealing, the height of these peaks increased, thus verifying crystal growth. The as-deposited XRD pattern shows a peak shift compared with the standard phase of the BaTiO_3_ cubic perovskite crystal; this shift can be attributed to the existing crystal lattice distortion and residual stress during AD. The thermal treatment of the film at 500 °C for 2 h relieved these defects and the peaks of the XRD pattern recovered to the standard phase. The accompanying 2 ${\rm{\mu }}{\rm{m}}\times$ 2 ${\rm{\mu }}{\rm{m}}$ AFM images depict the process of grain growth on the film surface^15^.

### Surface Morphology

**Figure 5:**
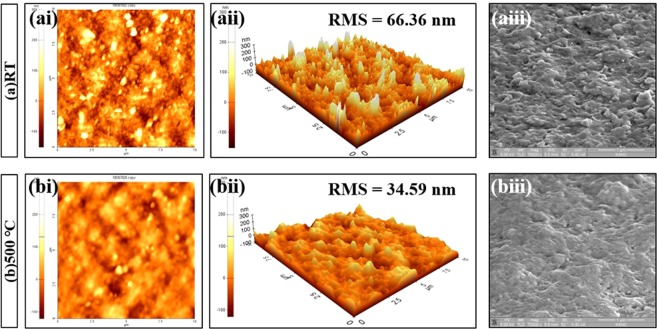
AFM graphs of the plan view, 3D surface view, and FIB graphs for the AD-prepared films. The film surface shows widely distributed BaTiO3 particles (ai) whereas less individual particles and pinholes are observed on the BaTiO3 films treated at 500 °C (bi). RMS decreases from 66.36 nm (aii) to 34.59 nm (bii) after thermal treatment at 500 °C. The as-deposited BaTiO3 film shows greater flatness (aiii) than the BaTiO3 film treated at 500 °C (biii).

The AFM and focused ion beam (FIB) images for RT films and films treated at 500 °C are shown in Fig. 5; from the figure, an increase in surface flatness owing to the decrease of the root mean square (RMS) from 66.36 nm to 34.59 nm is observed. Furthermore, the RT film surface shows many pinholes and weak particle-to-particle bonding; Hong-Ki Kim verified these defects as the primary sources of leakage current by scanning the dielectric surface using a conductive AFM^15^. The better flatness effect realized by annealing at 500 °C will effectively alleviate structural defects and ensure better dielectric performance. This indicates that the thermal treatment leads to the gradual repair of surface defects on the RT film. Because the RT film had several defects owing to strong powder impact, it was natural that this film had a rough surface. However, these defects can be reduced by the post annealing process because this auxiliary process helps to expand the grain size, thus filling the defects.

The severe fragmentation of the BaTiO_3_ film owing to the high-speed impact of the particles on the high-hardness glass substrate forms films with heterogeneous density and surface defects. The subsequent hammering effects lead to etching on the surface, especially, in the case of the partially fragmented regions. Hence, the as-deposited film surface typically features crater defects and pinholes. The variations in morphology owing to thermal treatment was caused by both internal solid-state reactions and previously-mentioned growth of surface grains, which lead to repair of severe surface defects and consequently result in better flatness^16,17^.

**Figure 6:**
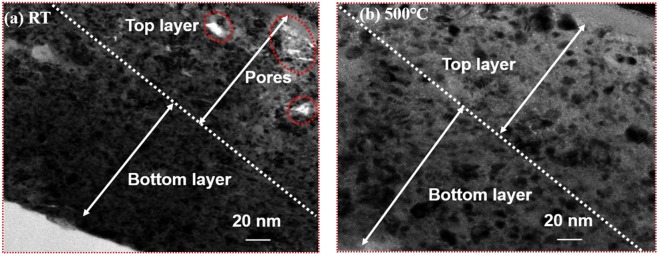
Cross-sectional views of the BaTiO3 films in transmission electron microscopy (TEM) graphs. (**a**) In the as-deposited films, there are several pores distributed on the top layer and the grains are of small size. (**b**) The thermal treatment at 500 °C eliminates the top-layer pores and the grains become larger. Moreover, the internal grain distribution becomes more homogeneous.

The cross-sectional TEM images of the BaTiO_3_ film before and after post-annealing are shown in Fig. 6. From the images, it is clear that there are many pores distributed on the top film layer at RT, whereas thermal treatment removes these pores and induces a more homogeneous cross-sectional structure. In particular, the post-annealing treatment at 500 °C resulted in clear grain growth and agglomeration, which filled up these holes, leading to a more homogeneous microstructure. In addition, the RT TEM image clearly shows a transitional-density side structure, which is caused by the accumulation of hammering effects. Relatively less hammering effects occurred at the top layer contribute in highly porous structure and thus low permittivity value. On the other hand, because the hammering effect was more at the bottom layer, it was more densely coated than the top layers. Therefore, the bottom layer had more stable dielectric layer than the top and middle layers. In the specific AD mechanism in our study, the inappropriate dielectric layer (top layer) caused the capacitance to be lower than the simulated value. On the other hand, these defects (pores) are filled with grain expansion in the annealing process, resulting in higher capacitance value. The loose grain-to-grain bonding and surface defects could also result in leakage current and overall low dielectric capacity^18^, which were improved by post-annealing, as previously mentioned, because post-annealing induces a solid-phase transformation from a non-uniform to uniform state. In the bottom layer, the thermal treatment promoted crystal growth and relieved the inner stress and crystal structure distortion, thus resulting in crystal property optimization. Simultaneously, the grain expansion by post-annealing also enhanced the grain-to-grain bonding, decreasing the number of inner pores, eventually leading to a more homogeneous internal structure^19,20^.

### Capacitance Values through Simulation and Measurement

**Figure 7:**
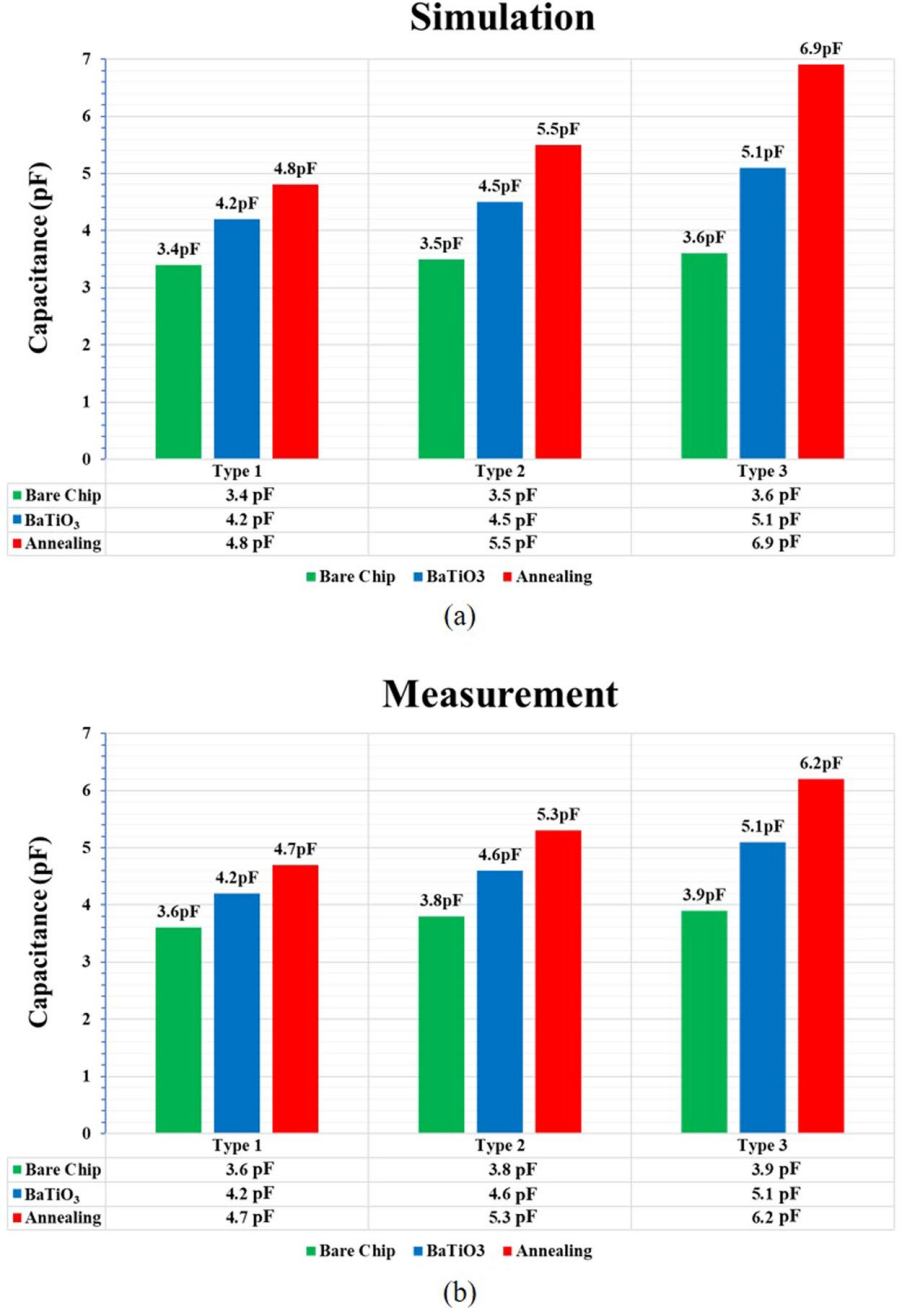
Three types of simulations and measurements performed in three different steps. The simulation results (**a**) show good consistency with the measured results (**b**). The BaTiO3 film deposited on the top of the IDC structures clearly enhances the capacitance whereas the post-annealing further improves the capacitance. The IDC pattern design also notably affects the capacitance, in which the type-3 design achieves higher capacitance than the other two designs.

Per our capacitance measurements, Type 3 circuits have higher capacitance than Type 1 and Type 2 circuits; this higher capacitance of Type 3 circuits might be attributed to higher coupling between electrodes owing to a narrower inter-width. The capacitances in the case of bare chips show only slight difference; however, these differences increase in the case of AD BaTiO_3_ films, especially after thermal treatment at 500 °C. In the case of annealed chips, the Type 3 circuit has a significantly higher capacitance than the rest. In addition, we calculated the dielectric constant and dielectric loss of the real glass substrate, BaTiO_3_ films, and annealed BaTiO_3_ films using the 1-MHz measurement results of the IDC chips. Our calculation results yield *ε* = 5.39*, tan δ = *0.00716; *ε* = 74.51*, tan δ = *0.04; and *ε* = 154.12*, tan δ = *0.01 for the glass substrate, BaTiO_3_ films, and annealed films respectively. By inputting these data as the parameter settings in ADS, the simulation results were agreed with the measured values Fig. 7(a)^21^.

From Fig. 7(a,b), it is clear that the annealing graph in the case of the Type 3 circuit shows a different value from the other two types. Furthermore, our evaluation results show that the Type 3 case suffered from thermal stress after the annealing process, which might be the reason that the actual measured capacitance is lower than that in the simulation results. We believe that the measured results will be similar to the simulation results if care is taken to avoid thermal stress during fabrication. In a future work, we might include the calculated dielectric constant and loss in the simulation software settings to enhance the obtained capacitance results, thus ensuring that they are closer to real-world scenarios.

In Table 3, we focus on three different types of IDCs and on the effects of the AD process and annealing temperature on the capacitance. The rate of increase of capacitance between bare chips and annealed chips can be defined as a simple equation.

5 $$\frac{Capacitance\,of\,annealed\,chip-Capacitance\,of\,bare\,chip}{Capacitance\,of\,bare\,chip}\times 100$$

Therefore, it can be concluded that the rate of increasing capacitance of the IDC chip with more finger number and narrow electrode width has efficient growth after annealing process. In Table 4, We have compared with other works with Type1–3 and concluded that having AD BaTiO_3_ annealing result comes out exceedingly better.

**Table 3:** Increase capacitance percentage in AD process and the annealing compare to bare chip.

Method	Type 1	Type 2	Type 3
AD Process	16%	20%	29%
Annealing at 500 °C	31%	39%	59%

**Table 4:** Comparison of capacitance performance with other works.

Works	Size	Frequency	Capacitance	Number of Fingers
^10^	0.09 × 0.15 mm^2^	<1 GHz	<1.7 pF	12
^22^	<8 × 8 mm^2^	1 MHz	2.34 pF	10
^23^	10 × 13.4 mm^2^	1 MHz	2.7 pF	18
Type 1	4.5 × 9 mm^2^	1 MHz	4.73 pF	19
Type 2	4.5 × 9 mm^2^	1 MHz	5.31 pF	24
Type 3	4.5 × 9 mm^2^	1 MHz	6.24 pF	32

## Conclusions

Three IDCs with an AD high-k dielectric layer were prepared and the differences between them were determined using simulations and measurements at a frequency of 1 MHz at room temperature. Bare IDCs, BaTiO_3_ IDCs at room temperature, and BaTiO_3_ AD IDCs annealed at 500 °C were manufactured with different finger widths and number of electrodes. The IDCs with a high number of electrodes showed higher coupling coefficient and stronger capacitive intensity. In addition, the thermal treatment on the AD film was shown to relieve the crystal structure distortion and internal stress, leading to better dielectric properties. Furthermore, in the case of the AD IDCs, the annealing Type 3 design showed an increasing capacitance percentage of 59% compare to bare chip. Hence, the optimized IDC structure with BaTiO_3_ films subjected to thermal treatment leads to capacitors with the highest performance values when compared with other thin layer capacitors. Therefore, they can be potentially used in super-humidity sensors, super-gas sensors, and super-biosensors as capacitive super-sensing applications in the future.
